# Transtelephonic Electrocardiographic Transmission in the Preparticipation Screening of Athletes

**DOI:** 10.1155/2008/217909

**Published:** 2007-11-25

**Authors:** Theodoros Samaras, Savvato Karavasiliadou, Evangelia Kouidi, John N. Sahalos, Asterios Deligiannis

**Affiliations:** ^1^Radiocommunications Laboratory, Department of Physics, Aristotle University of Thessaloniki, 54124 Thessaloniki, Greece; ^2^Laboratory of Sports Medicine, Department of Physical Education and Sport Science, Aristotle University of Thessaloniki, 54124 Thessaloniki, Greece

## Abstract

Transtelephonic electrocardiographic transmission (TET) is the most widespread form of telecardiology since it enables clinicians to assess patients at a distance. The purpose of this study was to assess the efficacy and effectiveness of TET either by fixed telephone line (POTS) or by mobile phone in the preparticipation screening of young athletes. A total of 506 players, aged 20.5 ± 6.2 years, from 23 soccer clubs in the prefecture of Thessaloniki, Greece, were physically examined in their playfields by a general practitioner (GP) and had their ECG recorded. In 142 cases, and on the judgment of the GP, the ECG was transmitted via POTS and/or global system for mobile communications (GSM) to a specialised medical centre where it was evaluated by a cardiologist. The mean total time for recording, storing, and transmitting the ECG was four minutes per subject. It was found that the success rate for transmission at first attempt was similar for both fixed and mobile networks, that is, 93% and 91%, respectively. The failure rate in the GSM network was correlated to the reception level at the site of transmission. Only in about half (*n* = 74) of the transmitted ECGs did the cardiologist confirm “abnormal” findings, although in 16, they were considered to be clinically insignificant. Consequently, 58 athletes were referred for further medical examination. Our results indicate that TET (either by fixed telephone line or by mobile phone) can ensure valid, reliable, and objective measurements, and significantly contribute to the application of medical screening in a great number of athletes. Therefore, it is recommended as an alternative diagnostic tool for the preparticipation screening of athletes living in remote areas.

## 1. INTRODUCTION

An increasing number of medical specialties such as dermatology, oncology, radiology, surgery, cardiology, and psychiatry use telemedicine routinely.In a country like Greece, which consists of several small islands dispersed around a mountainous mainland with many remote parts, telemedicine is not only beneficial but rather necessary for high-quality healthcare provision to its population.The services of telemedicine in their modern form appeared in Greece in 1991. Due to the low reliability of the plain old telephone service (POTS) at the beginning of these efforts [1], the implementations became wireless, via satellite links [2].However, the digitization of the POTS and the general improvement of the telecommunication infrastructure, especially the deployment of GSM (global system for mobile communications) networks, which took place since then, have favoured further developments in the field [3–5].

Telecardiology and specifically transtelephonic electrocardiographic transmission (TET) remains the most widespread form of telemedicine since it can ensure a valid, reliable, and objective assessment of cardiac rhythm and function.However, its use in sports medicine is not so widely mentioned, and relevant studies are very scarce in the international literature. Perhaps the only known application in this area is the follow-up of patients with cardiovascular disease during their rehabilitation exercise [6–10].

With the increasing number of individuals having physically active lifestyles, transient risk of cardiovascular events during exercise is not rare, especially among athletes with unrecognized cardiovascular diseases.Therefore, at least annual preparticipation screening is generally recommended prior to participation in sports.In order to increase the number of athletes properly tested, the screening program should be accurate, practical to apply under field conditions to large numbers of subjects, and have relatively low cost.

The purpose of the study was to evaluate whether it was technically feasible and effective for a general practitioner (GP) to examine amateur soccer players in their playfields and transmit, when this was judged necessary, a 12-lead ECG and a clinical record to a sports medicine base for further evaluation by a cardiologist. A secondary purpose was to determine whether the data could be reliably transmitted via the cellular communication system, as an alternative in the absence of a fixed telephone line.

## 2. METHODS

### 2.1. Study population and design

Ten percent of the active soccer clubs of the prefecture of Thessaloniki, in northern Greece, participated in this study. Specifically, from the total of 230 soccer clubs, 23 of urban and rural areas were randomly selected. The study population included 506 young soccer players, who were asked to fill in a questionnaire so that information about the medical record of the athlete and his family could be collected.Additionally, anthropometric and clinical data were recorded, that is, height, weight, systolic and diastolic blood pressure, and resting heart rate. All athletes underwent physical examination, including precordial auscultation in both the supine and standing positions. Murmurs were further evaluated with the Valsalva maneuver.Additionally, resting 12-lead ECG was performed in all subjects. In the case of unusual findings, as judged by the GP, from the medical history, physical examination and/or ECG, the latter together with a clinical record were transmitted from the examination site to the Laboratory of Sports Medicine for further evaluation by a cardiologist.

### 2.2. Telecardiology procedure

The device used for storing and transmitting the ECG was a portable electrocardiograph (Cardiette Excel 106scp) with an internal modem and a capability of connecting to an external one with a transmission speed of up to 19.2 kbps.An important feature of the electrocardiograph was the compression of data before transmission, which allowed high quality 12-lead recordings to be sent in small files. In the absence of a fixed telephone line (POTS), a dual-band GSM (900/1800 MHz) modem was used (Siemens TC35 Terminal) working with the circuit switched data (CSD) bearer service of a national operator with more than 99% geographical coverage and a nontransparent data rate of 9.6 kbps.The data calls from the electrocardiograph were terminated at a workstation running the software package Medibox Cardiobox of H&C Medical Devices S.p.A. and connected with a modem to the POTS.The communication between the cardiologist sitting in front of this workstation and the medical team in the field for reporting was always carried out with a mobile phone.Following the initial assessment, 58 athletes were given further appointments for echocardiography, stress test, and 24-hour Holter monitoring of ECG or blood pressure.

Descriptive statistics were used to describe categorical variables.For statistical analysis, the Statistics Toolbox of MATLAB was used (The Mathworks Inc., MA, USA). The correlation coefficient was derived to assess the association between the reception level of GSM and the number of failed transmissions. A *P* < .05 was accepted as statistically significant.

## 3. RESULTS

The locations where the preparticipation screening took place were at a distance between 4 and 82.2 km (19.35 ± 18.22 km) from the specialised examination centre at the Laboratory of Sports Medicine, which is situated at the center of the city of Thessaloniki. The mean transportation time of the healthcare team (one GP and one nurse) from the city centre to the locations of the athletes was 23.26 minutes (SD 17.73).

Most of the soccer clubs (*n* = 16 or 69.6%) had their own sports facilities, where the athletes were examined. However, only a small number (*n* = 5 or 21.7%) among them could offer an infirmary. Therefore, the screening was performed at other sites (e.g., offices, canteens, dressing rooms) for the remaining soccer clubs.

A fixed telephone line was available only at 17 sites (73.9%), whereas a GSM signal was present everywhere.

The physical characteristics of the 506 soccer players who were examined are shown in Table 1. Taking into account the importance of the family history in identifying athletes at risk of sudden death during sports, we found that 28.3% of the athletes had a family history of cardiovascular disease. Concerning their personal history, 8.1% of the athletes reported symptoms such as chest pain or discomfort during exercise. From the physical examination, mild (1/6) systolic murmurs were found in 15.2% and hypertension (brachial blood pressure >140/90 mmHg on >1 reading) in 0.4% of the athletes. A total of 142 ECGs were transmitted either by POTS (*n* = 108 or 76%) or GSM (*n* = 34 or 24%). ECG transmission on the first attempt was achieved in 93% and 91% of the cases, respectively. The ECG transmission was deemed unsuccessful in case of disconnects due to technical difficulties. The mean time for recording and storing the ECG was 180 s (SD 46). The transmission time was 60 seconds (SD 23) depending on the file size, which ranged between 6 and 11 kB. The above mean transmission time also includes repeated attempts.The mean connection time was 24 sesconds (SD 4).

The high rate of transmission failure was due to the low reliability of POTS connection or bad reception level in the case of GSM data calls (Table 2). Additionally, there was a weak but significant correlation coefficient between the reception level and the number of failed transmissions (*r* = −0.66, *P* < .005).

At the end of each day, the cardiologist at the Laboratory of Sports Medicine examined the records and the ECGs of the athletes tested during the day. There was no disagreement between the cardiologist and the GP on the ECGs that were not sent. With regard to the transmitted clinical records and ECGs, the cardiologist at the Laboratory of Sports Medicine, who had monitored and examined them, agreed only in 74 cases with the GP in the playfield on the need to have sent the ECGs due to the existence of suspicious findings. However, from the 74 cases, 16 athletes were not referred for further cardiovascular assessment as the cardiologist diagnosed that the findings were without clinical significance. From the remaining 58 cases, 49 athletes were referred for echocardiography, 7 were subjected to a stress test, and 2 to a 24-hour ambulatory Holter monitoring. After further evaluation, all of them were, eventually, permitted to participate in physical activities.

## 4. DISCUSSION

Our results indicate that telecardiology and specifically TET, interpreted by a sports medicine centre-based cardiologist, allows a rapid assessment of athletes at risk of adverse events during exercise, without the presence of a sports cardiologist.Thereby, qualitative care at lower cost can be provided to a large population and can improve compliance.It is also shown that utilizing the advancement of telecommunications, GSM transmission of ECG, is reliable and flexible, and can be used as an alternative when there are no facilities.

Several studies have already proven that TET is useful and reliable, and can provide early diagnostic information in cardiac patients with symptoms such as chest pain, dyspnea, palpitations, and so forth, [11–16]. So far, the cellular telephone communication system has been used to transmit 12-lead ECGs from ambulances to a hospital [3, 5], especially in case of chest pain [17]. However, this is the first study introducing TET in sports medicine and specifically in the preparticipation screening of athletes.

In this study, a store-and-forward transmission method was used.Alternatively, real-time transmission could have been used, which, however, would have required a larger bandwidth and would cost more.It should be mentioned here that wired broadband services are not widely available in Greece.Indeed, in 2005, about 67% of home Internet connections were still dial-up connections on the PSTN (public switched telephone network) [18].Among midband solutions, ISDN is more popular with 21% and ADSL is gaining in popularity (8%) very slowly, mainly due to the high prices.On the other hand, most mobile operators in Greece provide a general packet radio service (GPRS) with higher data transfer rates within urban areas, but a financial analysis must be done before one moves towards this solution, due to the different way of charging (per data volume, instead of calling time) and the initial cost of equipment.

It is worth comparing the results of the current study with a similar one in the literature, which was conducted in Athens, Greece. Kyriacou et al. [4] reported that the mean connection time between the telemedicine unit and the base unit was 28 seconds, a value that is very close to the one reported here. They also mention a success rate of 93% for the first attempt, which compares very well with the 91% of our study. One important difference between the two studies is the achieved data throughputs, which is about the double in Kyriacou et al. [4], but this could be attributed to the service used, since the authors mention the high speed circuit switched data (HSCSD), which offers high data transfer rates.Two more points are worth mentioning from our work: first, the reception level was correlated with the efficiency of the GSM data link and, second, the small mean total time needed to test each subject, that is, four minutes for recording, storing, and transmitting the ECG.

One shortcoming of the study, which needs to be addressed in its future follow-ups, was that it did not consider checking the accuracy of transmission. One way would have been for the cardiologist to have compared, at the end of the day, the received ECGs against their print-outs from the portable electrocardiograph and not just reviewing all the printed ECGs.

In conclusion, the use of telecardiology in the preparticipation screening of athletes was successful. It managed to distinguish the cases, which required further examination, and significantly reduced the logistics effort needed for the examination of all the subjects at the specialized medical centre. However, before it can be claimed that the procedure used in the present study can be generalized to other forms of preparticipation screening, a more careful cost-benefit analysis and an accuracy checking need to be performed.

## Figures and Tables

**Table 1:** Physical data of all soccer players examined (mean values ± SD).

Age (years)	20.5 ±6.2
Training duration (years)	10.8 ± 5.5
Training frequency (times/week)	3.4 ± 1.0
Height (cm)	175.0 ± 10.3
Weight (kg)	71.2 ± 12.1

**Table 2:** A number of unsuccessful transmissions with GSM. Attempts were repeated until successful transmission. Only 34 of the total attempted transmissions concerned actual patient ECGs. The rest of the transmissions involved ECGs saved in the memory of the portable electrocardiograph.

Location	Type	Reception level (dBm)	Number of total attempted transmissions	Number (percentage) of failed transmissions
Kordelio	Urban	−99	17	8 (47%)
Chalastra	Rural	−95	7	1 (14%)
Neochorouda	Rural	−91	10	5 (50%)
Mikra	Rural	−91	24	5 (21%)
Oreokastro	Suburban	−85	6	2 (33%)
Anatoliko	Rural	−85	4	0
Chalkidona	Rural	−83	5	1 (20%)
Vassilika	Rural	−83	6	2 (33%)
Liti	Rural	−81	5	1 (20%)
Asvestochori	Rural	−81	4	0
Pilea	Urban	−81	4	0
Evosmos	Urban	−79	4	0
Plagiari	Rural	−77	4	0
Krithia	Rural	−77	6	2 (33%)
Polichni	Urban	−77	4	0
Sikies	Urban	−75	6	0
Ampelokipi	Urban	−75	5	0
Diavata	Suburban	−71	7	3 (43%)
Ano Ilioupoli	Urban	−71	4	0
